# Multidimensional Poverty and Risk of Atherosclerotic Cardiovascular Disease: A U.S. National Study

**DOI:** 10.1016/j.jacadv.2024.100928

**Published:** 2024-05-15

**Authors:** Sara Ayaz Butt, Mauricio Tano Retamales, Zulqarnain Javed, Kobina Hagan, Hassaan Bin Arshad, Safi Khan, Isaac Acquah, Zlatko Nikoloski, Elias Mossialos, Miguel Cainzos-Achirica, Khurram Nasir

**Affiliations:** a Houston Methodist Research Institute, Houston Methodist, Houston, Texas, USA; b Nuclear Engineering Department, Texas A&M University, Texas, USA; c Houston Methodist DeBakey Heart & Vascular Center, Houston Methodist DeBakey Heart and Vascular Center, Houston, Texas, USA; d Department of Internal Medicine, MedStar Health, Baltimore, Maryland, USA; e London School of Economics and Political Science, London, United Kingdom; f Department of Cardiology, Hospital del Mar/Parc de Salut Mar, Barcelona, Spain; g Barcelona Biomedical Research Park, Barcelona, Spain

**Keywords:** cardiovascular disease, multidimensional poverty, poverty, social determinants of health

## Abstract

**Background:**

Poverty is associated with atherosclerotic cardiovascular disease (ASCVD). While poverty can be evaluated using income, a unidimensional poverty metric inadequately captures socioeconomic adversity.

**Objectives:**

The aim of the study was to examine the association between a multidimensional poverty measure and ASCVD.

**Methods:**

Survey data from the National Health Interview Survey was analyzed. Four poverty dimensions were used: income, education, self-reported health, and health insurance status. A weighted deprivation score (*c*_*i*_) was calculated for each person. The multidimensional poverty index was computed for various cutoffs, *k*, for total population, and by ASCVD status. The association between multidimensional poverty and ASCVD was examined using Poisson regression. Area under receiver operator characteristics curve analysis was performed to compare the multidimensional poverty measure with the income poverty measure as a classification tool for ASCVD.

**Results:**

Among the 328,164 participants, 55.0% were females, the mean age was 46.3 years, 63.1% were non-Hispanic Whites, and 14.1% were non-Hispanic Blacks. Participants with ASCVD (7.95%) experienced greater deprivation at each multidimensional poverty cutoff, *k*, compared to those without ASCVD. In adjusted models, higher burden of multidimensional poverty was associated with up to 2.4-fold increased prevalence of ASCVD (*c*_*i*_ = 0.25, adjusted prevalence ratio [aPR] = 1.66, *P* < 0.001; *c*_*i*_ = 0.50, aPR = 1.99; *c*_*i*_ = 0.75, aPR = 2.29; *P* < 0.001; *c*_*i*_ = 1.00, aPR = 2.38, *P* < 0.001). Multidimensional poverty exhibited modestly higher discriminant validity, compared to income poverty (area under receiver operator characteristics = 0.62 vs 0.58).

**Conclusions:**

There is an association between the multidimensional poverty and ASCVD. Multidimensional poverty index demonstrates slightly better discriminatory power than income alone. Future validation studies are warranted to redefine poverty's role in health outcomes.

Poverty is associated with cardiovascular disease (CVD) incidence, morbidity, and mortality.^1^, ^2^, ^3^ Traditionally, poverty has been evaluated using monetary approach, which measures it as a shortfall in income/consumption from some poverty line.^4^ However, poverty is a state in which people are exposed to ‘clustered disadvantages,^5^ including homelessness, unemployment, violence, and health catastrophes, among others. Hence, a unidimensional poverty metric inadequately captures the true extent of socioeconomic adversity experienced by individuals with CVD.

Amartya Sen’s capabilities approach presents an alternative paradigm capturing poverty's multidimensional nature, rejecting income as the sole measure of welfare, and emphasizing the expansion of capabilities for human development.^6^ It focuses on indicators that reflect the freedom to live a “valued life.” Poverty, in Sen’s framework, signifies the failure to achieve basic capabilities, which are crucial functionings at a minimally adequate level. Incorporating Sen's approach, multidimensional poverty measures encompassing monetary and nonmonetary dimensions of socioeconomic well-being can better identify individuals with CVD, surpassing income-based metrics. Given the substantial medical expenses associated with established CVD,^7^ such measures are crucially needed but currently lacking.

Alkire and Foster^8^ operationalized Sen’s theoretical framework through Alkire-Foster Counting methodology, which measures multidimensional poverty in national and global context. To the best of our knowledge, no large-scale, population-based studies in the United States have used the method to examine the association between a comprehensive multidimensional poverty index (MPI)—inclusive of income, education, insurance status, and self-reported health—and atherosclerotic CVD (ASCVD). To address this knowledge gap, we used the Alkire-Foster Counting methodology to: 1) compute multidimensional poverty estimates for the general population and in adults with and without ASCVD; 2) examine the association between multidimensional poverty and ASCVD; and 3) extend previously published analysis by Alkire and Foster^8^ from the year 2004 to the years 2007 to 2018 using the same data (National Health Interview Survey [NHIS]). We also compared the discriminatory ability of multidimensional poverty vs unidimensional income-based poverty for the classification of prevalent ASCVD.

## Methods

### Data Source and Study Design

This study used pooled data from respondents of the NHIS from 2007 to 2018.^9^ NHIS is an annual household interview survey of the noninstitutionalized U.S. civilian population conducted by the National Center for Health Statistics. Respondents, sampled using a complex multistage area probability design, report the following information: demographic and relationship information about all persons in the household (Household Composition Core); health status, health care access, and utilization for each family in the household (Family Core); and further information from a child and adult selected from each family (Sample Child and Sample Adult Cores). We used the NHIS Sample Adult Core file with supplementation of variables from Household Composition and the Family Core files. NHIS data are publicly available and deidentified; hence, this study was exempt from the purview of the Houston Methodist’s Institutional Review Board.

### Study Variables

#### Multidimensional poverty

Multidimensional poverty, estimated using the Alkire-Foster counting methodology (described in detail elsewhere^8^), was the primary independent variable of interest. It involves constructing a weighted deprivation score (c_i_) at the individual level, which is then used to calculate the MPI at the population level.

#### Weighted deprivation score (c_i_)

The Alkire-Foster method identifies a set of dimensions (d), which are different aspects of well-being that are important for a person's overall quality of life. An individual is said to experience poverty in a dimension if their dimensional achievement is below a specified deprivation cutoff level. Based on Alkire and Foster,^8^ we included income, education, self-reported health, and health insurance status as dimensions of poverty. Deprivation cutoffs were defined as household income below the federal poverty level, less than high school education, ‘fair’ or ‘poor’ self-reported health, and no health insurance, respectively. All dimensions were assigned an equal weight of ¼, representing their relative importance, such that the sum of all weights equals 1. The weighted deprivation score (c_i_) is computed by summing the weighted dimensions for each individual. The score reflects the proportion of simultaneous dimensional deprivations experienced by each person. Thus, our weighted deprivation score variable had values of 0 (no deprivation in any dimension), 0.25 (deprived in 1 dimension), 0.50 (deprived in 2 dimensions), 0.75 (deprived in 3 dimensions), and 1.00 (deprived in all 4 dimensions), respectively.

#### Multidimensional poverty index (MPI)

The MPI reflects the share of weighted disadvantages the poor encounter within a society, compared to overall potential disadvantages that would exist if all people were poor and were deprived in all dimensions. It is calculated as the product of the proportion of multidimensionally poor people (H) and the fraction of dimensions in which the average poor person is deprived (A). The multidimensionally poor individuals are identified based on a predetermined poverty cutoff (k), which represents the minimum weighted deprivation score. Individuals with a weighted deprivation score, c_i_, equal to or greater than ‘k’ were identified as multidimensionally poor.^8^ To calculate H, total number of multidimensionally poor people was divided by the population under study. To calculate A, the weighted deprivation scores of all individuals identified as multidimensionally poor were summed and divided by the total number of multidimensionally poor persons.^8^ A higher MPI score indicates greater dimensional deprivation among the average individuals in the studied population. Mathematical formulation of MPI is detailed in the Supplemental Appendix.

### ASCVD

ASCVD was the primary dependent variable. Respondents who reported ever receiving a diagnosis of angina pectoris, heart attack (myocardial infarction), coronary heart disease, or stroke were classified as having ASCVD, defined as a binary (yes/no) variable.

### Other Variables

Age, sex, race and ethnicity, cardiovascular risk factors, and major medical comorbidities were included as covariates. All covariates were self-reported and categorized as used in analyses. Race and ethnicity were categorized as non-Hispanic White, non-Hispanic Black, Hispanic, Asian, and others. The NHIS uses self-identification to document information on race and ethnicity.^10^ Cardiovascular risk factors included hypertension, diabetes mellitus/prediabetes, smoking, obesity, high cholesterol, and insufficient physical activity (≤150 minutes per week of moderate intensity aerobic physical activity or ≤75 minutes per week of vigorous intensity aerobic physical activity). Comorbidities relevant to the study included emphysema, chronic obstructive pulmonary disease, asthma, gastrointestinal ulcer, any cancer, arthritis, hepatitis/any liver conditions, and chronic kidney disease. Inflation-adjusted income-poverty threshold ratio variable published annually in the NHIS datasets, was used as a measure of income poverty. The variable is constructed by taking the ratio of annualized household income value reported by the respondents to the income poverty threshold for the survey year, given information on the household size. An individual was considered income poor if the ratio of their annual household income to federal poverty level was ≤1.00.

### Statistical Analysis

We included respondents ≥18 years of age for whom complete information on all-dimensional achievements was available in the survey. Consequently, 29,550 (8.26%) of observations were dropped from analysis. Missing data for dimensional achievement was as follows: income 7.54%, education 0.50%, health insurance 0.55%, and self-reported health 0.05%. Since the distribution of study characteristics between individuals with complete information on all 4 dimensions and those with any missing information was similar, we did not impute any missing values. Supplemental Table 1 presents comparison of characteristics of participants included vs excluded from the study.

We reported summary statistics of the demographic and clinical characteristics for the total study population (ie, all NHIS 2007-2018 participants meeting the study inclusion criteria) and for persons with and without ASCVD separately. Chi-squared tests were used to compare differences between ASCVD and non-ASCVD population subgroups. We estimated MPI, H, and A for the total study population as well as by ASCVD status, using 4 poverty cutoffs: *k* (0.25, 0.50, 0.75, and 1.00). All multidimensional poverty estimates along with their measures of accuracy and *P* values were obtained using the Bootstrap methodology with 1,000 replications of sampling with data replacement.

The weighted deprivation score *c*_*i*_ was defined as a measure for multidimensional poverty burden among individual participants. We used Poisson regression to generate prevalence ratios (PR) of the association between multidimensional poverty and prevalent ASCVD along with *P* values and 95% CI. Poisson regression with robust variance provides correct estimates in cross-sectional studies, where exposure and outcome are measured at the same point in time.^11^ Three models were generated: an unadjusted model (Model 1); a model adjusted for age, sex, and race and ethnicity (Model 2); and a fully adjusted model (Model 3) including cardiovascular risk factors, comorbidities, and all variables in Model 2.

We compared the discriminant validity of multidimensional poverty with income-based poverty using receiver-operating characteristics (ROC) curves. The ROC curve for multidimensional poverty was plotted using the weighted deprivation score variable, while that for income poverty was plotted using the income-poverty threshold ratio.^12^

In additional analysis, we decomposed MPI estimate obtained at k = 0.50^8^ by race and ethnicity for participants with and without ASCVD to understand potential variation in multidimensional poverty characteristics for these population subgroups. Decomposition method is described in detail elsewhere.^8^^,^^13^ We also calculated percentage contribution of each race and ethnicity subgroup poverty to overall poverty (% contribution = subgroup population share · subgroup MPI/overall MPI). Next, we decomposed the poverty within race and ethnicity down by dimensions to examine how different race and ethnicity groups with and without ASCVD have different dimensional deprivations^13^. We calculated the censored headcount ratio of each dimension, h_*j*_(*k*), (percentage of population who are both multidimensionally poor and simultaneously deprived in that dimension) and then computed the percentage contribution of each dimension to MPI (% contribution = w_*j*_ · h_*j*_(*k*)/MPI)^13^. Finally, to observe robustness of rank ordering of MPIs obtained for race and ethnicity groups to changes in k, we estimated MPI for various values of *k* for both ASCVD and non-ASCVD groups.

The data were weighted to obtain nationally representative estimates. Variance estimation for the entire pooled cohort was obtained from the Integrated Public Use Microdata Series. For all statistical analyses, a 2-tailed *P* < 0.05 was considered statistically significant, and 95% CI was used to evaluate the precision of estimates. All analyses were performed using Stata, version 16 (StataCorp, LP).

## Results

### Study Population

The study sample comprised 328,164 adults, representing 216.3 million annualized U.S. adults, with complete data on dimensional achievements.

### Participant Characteristics

The demographic and clinical characteristics of the study population are presented in Table 1, overall and by ASCVD status. Mean age was 46.3 ± 17.6 years; 55% participants were women; 14.1% were non-Hispanic Black; and 16.1% were Hispanic. In total, 7.95% of participants self-reported having ASCVD. Compared to participants without ASCVD, those with ASCVD were more likely to be older, male, non-Hispanic Black or Hispanic, experience poor cardiovascular risk profile (27.3% vs 6.06%), and report at least 2 comorbidities (41.1% vs 11.7%).

### MPI Distribution at the Population Level

Table 2 presents the population-level distribution of MPI for the overall study population as well as by ASCVD status for various values of multidimensional poverty cutoff (k). We observed that as *k* increased from 0.25 to 1, as expected, the headcount ratio of multidimensional poverty, *H*, decreased from 40.0% to 0.39%, while the intensity of multidimensional poverty experienced, *A*, increased from 37.5% to 100%. For all values of *k*, persons with ASCVD experienced a greater burden of multidimensional poverty compared to the non-ASCVD group (*k* = 0.25, MPI: 0.236 vs 0.141; *k* = 0.50, MPI: 0.153 vs 0.082; *k* = 0.75, MPI: 0.061 vs 0.030; *k* = 1.00, MPI: 0.005 vs 0.004). The difference between MPI estimates for the subgroups with and without ASCVD was statistically significant for all values of *k*. This difference is largely driven by greater values of *H* in the ASCVD group for all values of *k* (*k* = 0.25, *H*: 59.5% vs 38.1%; *k* = 0.50, *H*: 26.4% vs 14.2%; *k* = 0.75, *H*: 8.02% vs 3.91%; *k* = 1.00, *H*: 0.50% vs 0.38%). The headcount ratios (H) also show that a higher proportion of the ASCVD population is considered multidimensionally poor compared to the non-ASCVD population, regardless of the poverty cutoff. This suggests that ASCVD may be associated with a greater prevalence of poverty-related factors. The intensity of poverty (A) is relatively similar between the ASCVD and non-ASCVD populations, especially at lower poverty cutoffs. This implies that while the prevalence of poverty is higher in the ASCVD population, the severity of poverty experienced by the poor in both groups is comparable.

**Table 1:** Characteristics of Participants From the National Health Interview Survey 2007-2018

	Total Population (N = 328,164)	ASCVD (n = 30,144)	Non-ASCVD (n = 298,020)
Weighted sample	216,288,449	17,189,774 (7.95)	199,098,675 (92.1)
Age, y			
Mean ± SD	46.3 ± 17.6	64.6 ± 14.3	44.7 ± 17.0
18-39	117,655 (35.9)	1,425 (4.73)	116,230 (39.0)
40-64	138,821 (42.3)	11,279 (37.4)	127,542 (42.8)
65-74	40,860 (12.5)	8,201 (27.2)	32,659 (11.0)
≥75	30,828 (9.39)	9,239 (30.7)	21,589 (7.24)
Sex			
Male	147,734 (45.0)	15,661 (52.0)	132,073 (44.3)
Female	180,430 (55.0)	14,483 (48.1)	165,947 (55.7)
Race and ethnicity			
Hispanic	52,727 (16.1)	3,141 (10.4)	49,586 (16.6)
Non-Hispanic Asian	18,802 (5.73)	916 (3.04)	17,886 (6.00)
Non-Hispanic Black	46,103 (14.1)	4,589 (15.2)	41,514 (13.9)
Non-Hispanic White	206,903 (63.1)	21,122 (70.1)	185,781 (62.3)
Cardiovascular risk profile			
Optimal	164,445 (55.0)	4,880 (18.9)	159,565 (58.1)
Average	122,731 (37.2)	15,265 (53.8)	107,466 (35.9)
Poor	28,503 (7.71)	8,265 (27.3)	20,238 (6.06)
Comorbidities			
0	117,113 (59.1)	4,809 (25.8)	112,304 (62.1)
1	58,549 (26.8)	6,555 (33.1)	51,994 (26.2)
≥2	34,111 (14.7)	8,550 (41.1)	25,561 (11.7)

Values are n, mean ±, or n (%).

Chi-square test (t-test for mean age): *P* < 0.05 for covariate distribution across ASCVD and non-ASCVD subgroups, for all study variables.

ASCVD = atherosclerotic cardiovascular disease.

**Table 2:** Population-Level MPI Estimates, Overall and by ASCVD Status, for Various Poverty Cutoffs

	Total Population (N = 328,164)			ASCVD (n = 30,144)			Non-ASCVD (n = 298,020)			
	Estimate^a^	Value	95% CI^c^	Estimate^a^	Value	95% CI^c^	Estimate^a^	Value	95% CI^c^	*P* Value^b^
k = 0.25	MPI	0.150	0.149-0.151	MPI	0.236	0.233-0.239	MPI	0.141	0.141-0.142	<0.001
	H	40.0%	39.9%-40.2%	H	59.5%	58.9%-60.0%	H	38.1%	37.9%-38.2%	
	A	37.5%	37.4%-37.6%	A	39.7%	39.4%-39.9%	A	37.1%	37.0%-37.2%	
k = 0.50	MPI	0.0883	0.0876-0.0890	MPI	0.153	0.150-0.156	MPI	0.0817	0.0811-0.0824	<0.001
	H	15.3%	15.2%-15.4%	H	26.4%	25.9%-26.9%	H	14.2%	14.1%-14.3%	
	A	57.6%	57.5%-57.7%	A	58.1%	57.8%-58.3%	A	57.6%	57.4%-57.7%	
k = 0.75	MPI	0.0332	0.0326-0.0337	MPI	0.0614	0.0591-0.0637	MPI	0.0303	0.0298-0.0308	<0.001
	H	4.29%	4.22%-4.36%	H	8.02%	7.72%-8.32%	H	3.91%	3.85%-3.98%	
	A	77.3%	77.2%-77.4%	A	76.6%	76.3%-76.8%	A	77.4%	77.3%-77.5%	
k = 1.00	MPI	0.00391	0.00369-0.00412	MPI	0.005	0.004-0.00577	MPI	0.00380	0.00359-0.00401	<0.001
	H	0.391%	0.369%-0.412%	H	0.498%	0.418%-0.577%	H	0.380%	0.359%-0.401%	
	A	100.0%	-	A	100.0%	-	A	100.0%	-	

A = multidimensional poverty intensity; ASCVD = atherosclerotic cardiovascular disease; H = multidimensional poverty headcount ratio; k = multidimensional poverty cutoff; MPI = multidimensional poverty index.

The multidimensional poverty cutoff (k) is a threshold used to determine poverty status in the calculation of the multidimensional poverty index, based on the Alkire-Foster method. It represents the minimum number of weighted deprivations required across various indicators to classify an individual as multidimensionally poor. The poverty cutoff (k) values, such as k = 0.25 indicates deprivation in at least 25% of the indicators, k = 0.50 signifies deprivation in at least 50% of the indicators, and so on. If an individual’s weighted deprivation score (ci) is equal to or greater than poverty cutoff (k), they are classified as multidimensionally poor.

a Estimates are significant at *P* < 0.001.

b *P* value reported for the test of significance of difference (t-test) of MPI estimates for ASCVD and non-ASCVD population subgroups.

c Bootstrap method was used to obtain 95% CI for MPI and *H* & *A* estimates.

### Associations Between Individual-Level Multidimensional Poverty and ASCVD

Table 3 presents results from Poisson regression models. In fully adjusted models, greater severity of multidimensional poverty, as denoted by c_i,_ was associated with an incrementally higher prevalence of ASCVD. For instance, compared to those not deprived in any dimension (c_i_ = 0), persons deprived in only 1 dimension of poverty (c_i_ = 0.25) had 66% higher prevalence of ASCVD (PR = 1.66; 95% CI: 1.60-1.73); 99% higher prevalence for those deprived in 2 dimensions (c_i_ = 0.50) (PR = 1.99; 95% CI: 1.89-2.09); and 129% higher prevalence for those deprived in 3 dimensions (c_i_ = 0.75) (PR = 2.29; 95% CI: 1.84-3.07). Those deprived in all dimensions had a 2.4-fold higher ASCVD prevalence (PR = 2.38; 95% CI: 1.84-3.07).

**Table 3:** Association Between the Multidimensional Poverty Weighted Deprivation Score (*c*_*i*_) and Prevalent ASCVD

	Model 1^a^		Model 2^b^		Model 3^c^	
Multidimensional Poverty	PR (95% CI)	*P* Value	PR (95% CI)	*P* Value	PR (95% CI)	*P* Value
c_i_ = 0	Reference		Reference		Reference	
c_i_ = 0.25	2.12 (2.05-2.19)	<0.001	2.22 (2.15-2.29)	<0.001	1.66 (1.60-1.73)	<0.001
c_i_ = 0.50	2.58 (2.48-2.68)	<0.001	2.90 (2.80-3.01)	<0.001	1.99 (1.89-2.09)	<0.001
c_i_ = 0.75	2.88 (2.71-3.06)	<0.001	3.66 (3.45-3.88)	<0.001	2.29 (2.13-2.47)	<0.001
c_i_ = 1.00	2.08 (1.71-2.54)	<0.001	3.36 (2.77-4.08)	<0.001	2.38 (1.84-3.07)	<0.001

ASCVD = atherosclerotic cardiovascular disease; PR = prevalence ratios; ci = multidimensional poverty weighted deprivation score.

The multidimensional weighted deprivation score (ci) is computed using the Alkire-Foster method. This method considered dimensions such as income, education, self-reported health, and health insurance status to capture poverty. Each dimension was assigned an equal weight of 1/4, denoting their relative importance. The weighted deprivation score was obtained by summing the products of the weights and dimensional deprivations for each person. Hence, the ci represents the weighted proportion of simultaneous deprivations across multiple dimensions of poverty experienced by each individual. It has values of 0 (no deprivation in any dimension), 0.25 (deprived in any 1 dimension), 0.50 (deprived in 2 dimensions), 0.75 (deprived in 3 dimensions), and 1.00 (deprived in all 4 dimensions), respectively.

a Model 1 = Unadjusted.

b Model 2 = Adjusted for age, sex, and ethnicity/race.

c Model 3 = Adjusted for Model 2 + cardiovascular risk factors profile + comorbidities.

### Discriminant Validity of the MPI

**Figure 1:**
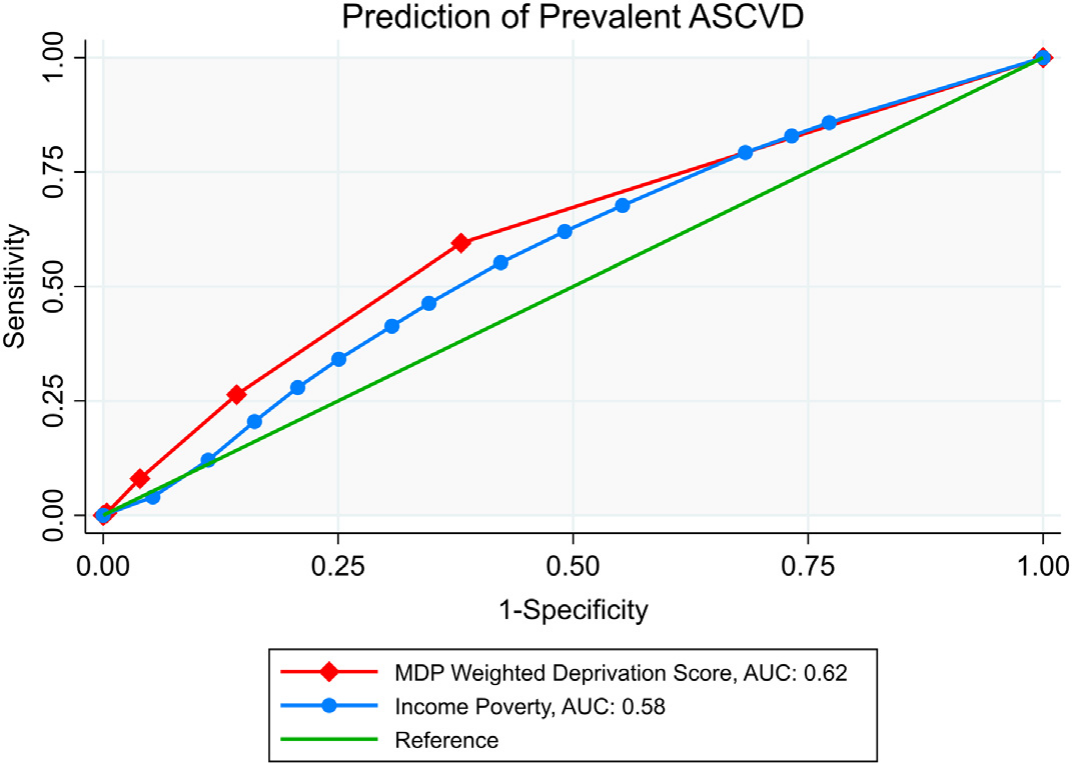
**Analysis of Discriminative Ability (AUROC) for Prediction of Prevalent ASCVD** The multidimensional weighted deprivation score (c_i_) is computed using the Alkire-Foster method. This method considered dimensions such as income, education, self-reported health, and health insurance status to capture poverty. Each dimension was assigned an equal weight of 1/4, denoting their relative importance. The weighted deprivation score was obtained by summing the products of the weights and dimensional deprivations for each person. The score ranges from 0 (no deprivation) to 1 (deprivation in all dimensions), indicating the weighted proportion of simultaneous dimensional deprivations experienced by each individual. ASCVD = atherosclerotic cardiovascular disease; AUC = area under curve; AUROC = area under receiver operator characteristics; MDP = multidimensional poverty.

Figure 1 (ROC) compares the performance of the multidimensional poverty measure in classifying ASCVD relative to income poverty. The area under curve for multidimensional poverty (0.62 ± 0.002) was modestly higher than that for the income poverty (0.58; S.E: 0.002), suggesting potentially higher discriminant validity of the former (versus the latter) as an ASCVD classification tool.

### Distribution of MPI by Race and Ethnicity Subgroups and ASCVD Status

Table 4 presents distribution of MPI by race and ethnicity for individuals with and without ASCVD sub-groups at a poverty cutoff of 0.5. The data reveals significant disparities in MPI across different racial and ethnic groups. Overall, regardless of ASCVD status, the degree of multidimensional poverty (MPI) was greatest among Hispanic persons followed by non-Hispanic Black individuals. Despite the relatively small proportion of Hispanic and non-Hispanic Black population in the total population (ASCVD: 10.4% and 15.2%; non-ASCVD: 16.6% and 13.9%), both of these subgroups contribute disproportionately higher toward MPI (ASCVD: 21.5% and 25.0%; non-ASCVD: 40.4% and 20.8%).

**Table 4:** Distribution of Multidimensional Poverty Index by Race and Ethnicity by ASCVD Subgroups at Poverty Cutoff, k = 0.50

Group	Population (N)	% Contribution	MPI^a^	95% CI^b^	% Contribution
ASCVD (n = 30,144)					
Hispanic	3,141	10.4%	0.316	0.302-0.330	21.5%
NH Asian	916	3.04%	0.154	0.135-0.174	3.10%
NH Black	4,589	15.2%	0.252	0.241-0.262	25.0%
NH White	21,122	70.1%	0.107	0.103-0.111	48.9%
Other	376	1.25%	0.184	0.154-0.214	1.50%
Total	30,144	100.0%	0.153	0.149-0.157	100.0%
Non-ASCVD (n = 298,020)					
Hispanic	49,586	16.6%	0.19	0.195-0.202	40.4%
NH Asian	17,886	6.00%	0.0504	0.047-0.054	3.70%
NH Black	41,514	13.9%	0.122	0.119-0.125	20.8%
NH White	185,781	62.3%	0.044	0.0432-0.0449	33.5%
Other	3,253	1.09%	0.113	0.100-0.120	1.50%
Total	298,020	100.0%	0.0817	0.0808-0.0826	100.0%

ASCVD = atherosclerotic cardiovascular disease; H = multidimensional poverty headcount ratio; MPI = multidimensional poverty index; NH = non-Hispanic.

Poverty cutoff, k = 0.50.

a Estimates are significant at *P* < 0.001.

b Bootstrap method was used to obtain 95% CI for MPI estimates.

**Figure 2:**
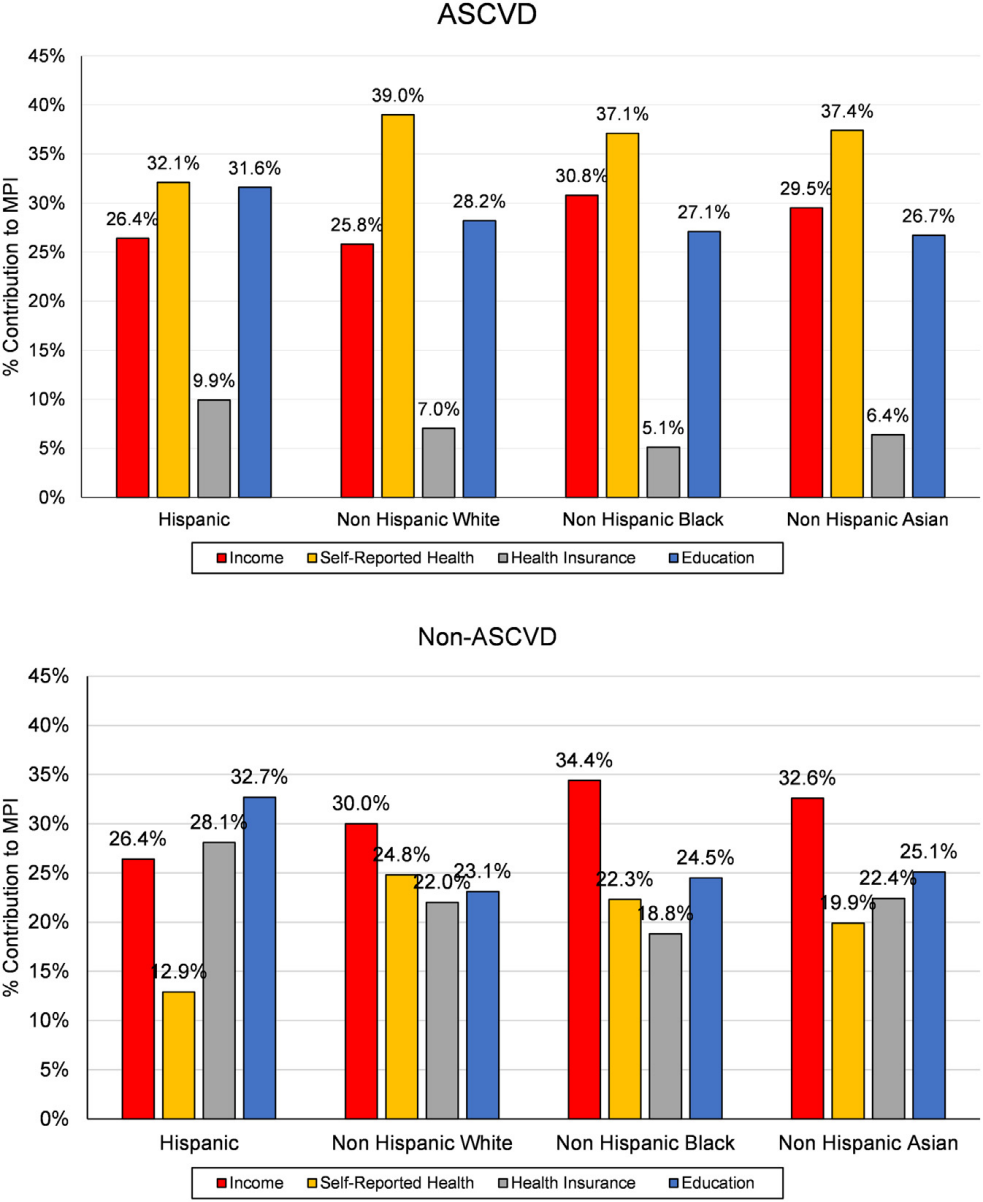
**Dimensional Deprivation Decomposition of MPI by Poverty Dimensions** ASCVD = atherosclerotic cardiovascular disease; MPI = multidimensional poverty index.

Figure 2 illustrates the dimensional breakdown of the MPI for each race and ethnicity subgroup for adults with and without ASCVD. These results show that the distribution of the percentage contribution of each dimension to the MPI varies across different race and ethnicity subgroups. In general, self-reported health and education tend to have higher contributions to the MPI, especially among adults with ASCVD, whereas income was the primary contributor in the non-ASCVD population. This pattern was observed for all racial/ethnic groups, except for the higher MPI contribution of education (versus income) for Hispanic adults without ASCVD. This indicates that addressing disparities in these dimensions could be crucial in reducing multidimensional poverty and its impact on ASCVD risk across different racial and ethnic populations.

**Figure 3:**
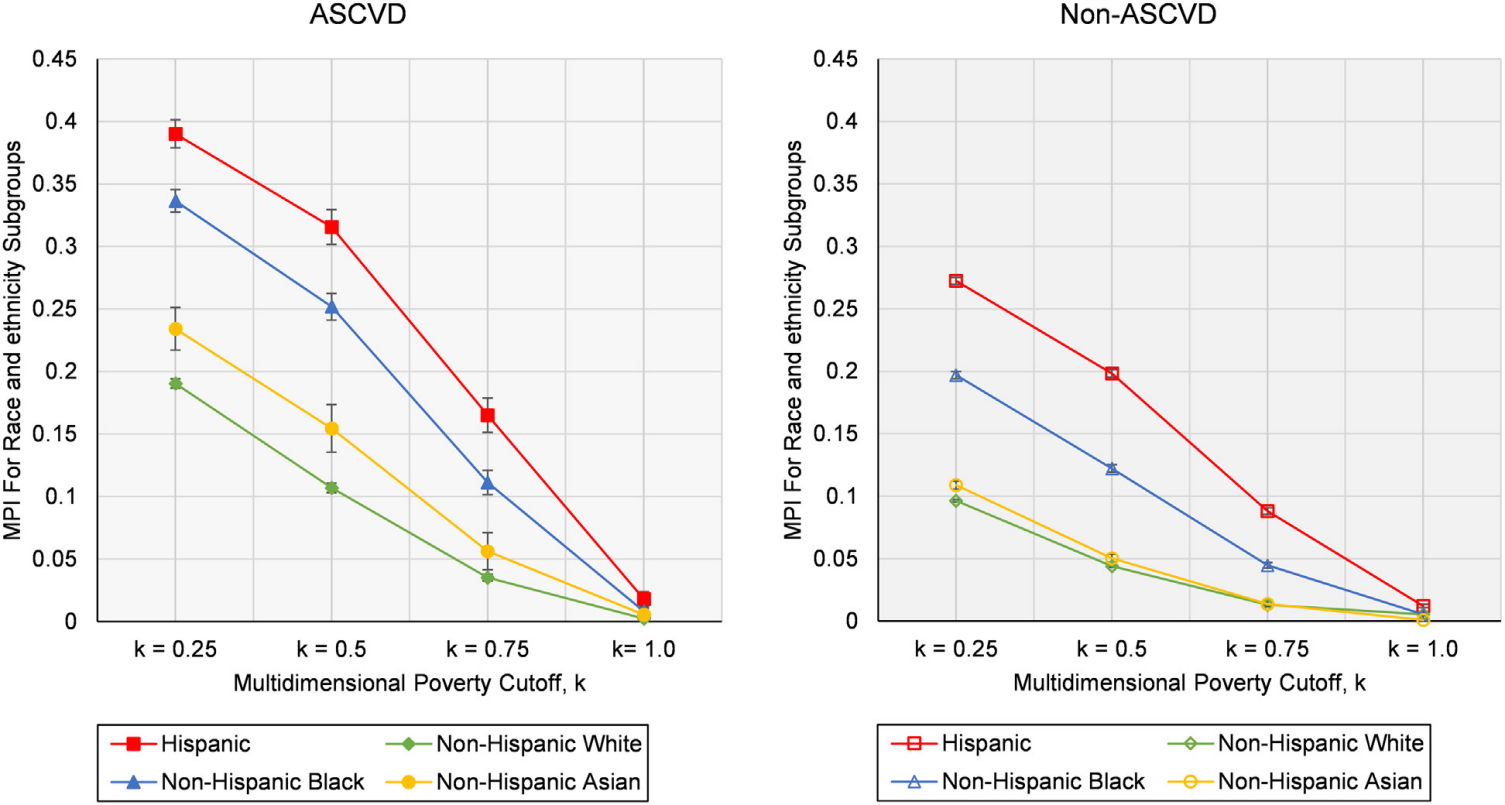
**Multidimensional Poverty Index (MPI) for Race and Ethnicity Groups by Poverty Cutoff, k** The multidimensional poverty cutoff (k) is a threshold used to determine poverty status in the calculation of the multidimensional poverty index, based on the Alkire-Foster method. It represents the minimum number of weighted deprivations required across various indicators to classify an individual as multidimensionally poor. The poverty cutoff (k) values, such as k = 0.25 indicates deprivation in at least 25% of the indicators, k = 0.50 signifies deprivation in at least 50% of the indicators, and so on. If an individual’s weighted deprivation score (c_i_) is equal to or greater than poverty cutoff (k), they are classified as multidimensionally poor. ASCVD = atherosclerotic cardiovascular disease; k = multidimensional poverty cutoff; MDI = multidimensional poverty index.

Figure 3 reports MPI levels computed by race and ethnicity subgroups for all values of k for participants with and without ASCVD. For any given poverty cutoff (k), individuals with ASCVD experience greater burden of multidimensional poverty compared to those without ASCVD. Additionally, consistent with the findings reported above, Hispanic and non-Hispanic Black individuals experienced higher burden of multidimensional poverty at any given poverty cutoff relative to non-Hispanic White individuals for both ASCVD and non-ASCVD populations, except for k = 1.00 (owing to the low sample size for this category). The distribution of multidimensional poverty by individual MPI dimensions and association with ASCVD is depicted in the Central Illustration.

**Central Illustration:**
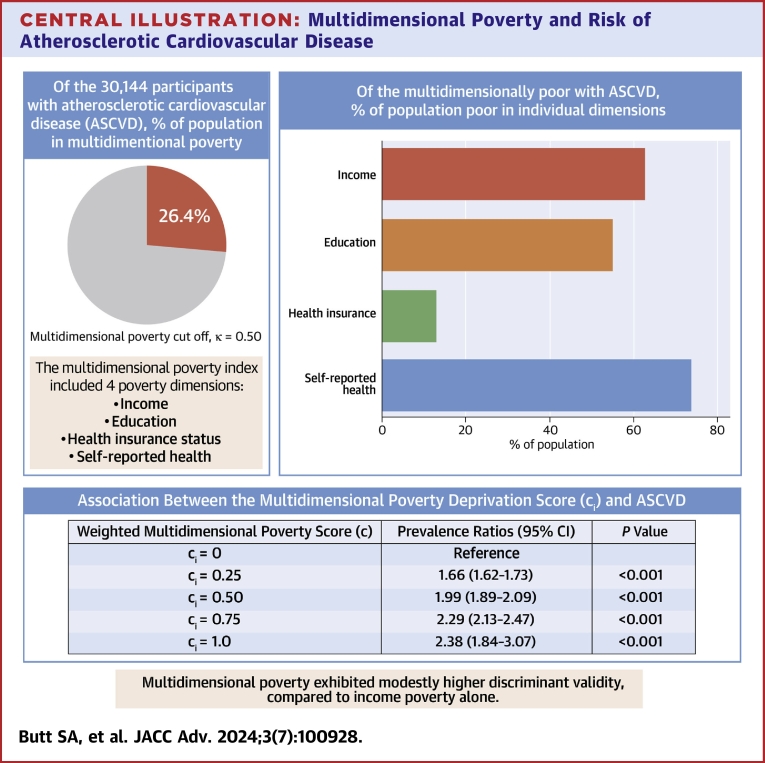
**Multidimensional Poverty and Risk of Atherosclerotic Cardiovascular Disease** A higher burden of multidimensional poverty is associated with up to 2.0-fold increased prevalence of ASCVD. The multidimensional weighted deprivation score (ci) considers dimensions such as income, education, self-reported health, and health insurance status to capture poverty. The ci has values from 0 to 1: 0 (no deprivation in any dimension), 0.25 (deprived in any 1 dimension), 0.50 (deprived in 2 dimensions), 0.75 (deprived in 3 dimensions), and 1.00 (deprived in all 4 dimensions). ASCVD = atherosclerotic cardiovascular disease.

## Discussion

In this nationally representative study, we quantified multidimensional poverty using Alkire-Foster Counting methodology in the United States and reported its association with ASCVD. We found that individuals with ASCVD experienced significantly greater multidimensional poverty burden, measured by the MPI, compared to non-ASCVD individuals, regardless of the poverty cutoff chosen. In multivariable analyses, we observed that a higher burden of simultaneous deprivations in poverty dimensions was associated with a higher prevalence of ASCVD, independent of clinical and demographic factors. Moreover, multidimensional poverty was found to have modestly higher discriminatory power for ASCVD classification compared to the unidimensional income poverty measure. Additionally, substantial disparities in multidimensional poverty across race and ethnicity subgroups were identified, with Hispanic and non-Hispanic Black individuals experiencing a greater burden of poverty than non-Hispanic White individuals, regardless of ASCVD status.

Our results are an important addition to the limited contemporary discourse on poverty in the CVD space. Existing literature is replete with traditional income-based measure of poverty and its inverse relationship with CVD risk factors and outcomes,^3^^,^^14^^,^^15^ or with reports of association between other individual dimensions of MPI (self-reported health,^16^, ^17^, ^18^ insurance,^19^ educational attainment)^1^^,^^2^^,^^14^ and CVD outcomes. However, no large-scale study in the U.S. has examined the aggregated effect of the independent social risk factors on ASCVD using a robust composite index of poverty that captures monetary and nonmonetary dimensions. One notable mention is Callander et al^20^ who constructed the ‘Freedom Poverty Measure’ and analyzed multiple forms of disadvantage experienced by those with no health condition, CVD, and all other health conditions in a cross-sectional study design. The Freedom Index was designed to assess the impact of government policy on the well-being of Australians, which may limit its applicability to the broader study of ASCVD outcomes and racial/ethnic disparities in different populations. The novelty of our study is the application of Alkire-Foster MPI, a validated global measure, to assess the association of multidimensional poverty with prevalent CVD on a large population scale in the United States. The MPI framework is flexible, allowing for the inclusion of context-specific indicators, but it also maintains a core set of dimensions, such as health, education, and living standards, that are relevant and comparable across different populations.

Furthermore, our study delved deeper into the disparities among different racial and ethnic groups in terms of multidimensional poverty and its impact on ASCVD outcomes. By providing a detailed breakdown of MPI values and percentage contributions for different racial/ethnic groups within ASCVD and non-ASCVD populations, our research has highlighted the inequities that exist among these groups and their potential influence on ASCVD outcomes. In the context of disparities research, these findings highlight the importance of considering multidimensional poverty as a key factor driving health disparities. Future studies should further explore the interplay between race and ethnicity, social determinants, and health outcomes to inform effective interventions. For public health policy, the results underscore the need to prioritize health equity by addressing systemic barriers that contribute to these disparities. Policymakers should collaborate with health care providers, community organizations, and other stakeholders to develop culturally sensitive, targeted programs addressing income, education, and health care access.

This paper demonstrates that greater socioeconomic adversity, highlighted by higher multidimensional poverty burden, was significantly associated with a higher prevalence of ASCVD. Our finding is consistent with recent evidence that looked at socioeconomic indicators, other social determinants of health (SDOH), and CVD risk factors and outcomes.^21^^,^^22^ Differences in income and other socioeconomic determinants are consistently linked to poor cardiovascular health and are major drivers of racial/ethnic disparities in CVD.^3^ Various physiologic, psychosocial, and behavioral mechanisms have been examined in scientific literature to establish biological plausibility for the observed associations between poor socioeconomic markers and adverse CVD outcomes.^21^^,^^23^ Hence, this calls for greater focus on addressing these and related SDOH in the ASCVD population through targeted policy interventions to mitigate disease burden. Further, the interdependent nature of individual dimensions of poverty (such as income, education, and financial burden of health care) has been demonstrated extensively in the literature.^24^^,^^25^ In this study, we reported the effects of individual poverty domains on ASCVD; future work should build on these findings and further explore intersectionality among the individual poverty domains to affect the risk of CVD.

Our study has important clinical implications as it highlights the need for a holistic approach to patient care that considers medical factors and SDOH, such as income, education, and self-reported health. Clinical practice should consider incorporating targeted interventions for at-risk populations facing multidimensional poverty and higher ASCVD risk, including culturally sensitive education and improved health care access. The MPI can be used as a tool to “flag” socially vulnerable individuals experiencing the negative consequences of socioeconomic inequities in the health care system. Furthermore, hospitals and health care providers should work toward designing cross-sectoral collaboration with social services, education, and community organizations to address broader health disparities and create comprehensive support systems that address persistent structural barriers.^26^ Prioritizing health equity in clinical practices, addressing implicit biases, and providing cultural competency training can ensure equal access to quality care for all patients. Hospitals should invest in continuous education and training for their health care providers, ensuring that they are up-to-date on the latest research and best practices for addressing multidimensional poverty and its impact on ASCVD. This can help ensure that providers are equipped to deliver the most effective care possible to their patients. Finally, leveraging data from studies like this can inform decision-making processes, helping to design and implement effective interventions to reduce health disparities and improve patient outcomes.

Our approach may be used to develop and validate similar tools in diverse sociodemographic and clinical subpopulations, and to assess their effects on other leading clinical outcomes, including CVD mortality and hospitalization. Our methodology and findings may inform future work to predict incident outcomes, including new-onset CVD, CVD mortality, and all-cause mortality, in diverse patient populations using longitudinal study designs.

### Study Limitations

This study is not without limitations. First, the cross-sectional nature of NHIS precludes an assessment of causal relationship between multidimensional poverty and ASCVD. Reverse causality between poverty and ASCVD cannot be definitively assessed in this study. Longitudinal research designs would be valuable in examining how changes in poverty dimensions over time impact the risk of CVD. Second, our findings are based on self-reported data, which may be subject to recall and reporting biases. While the NHIS undergoes quality checks and has demonstrated good correlation with clinically ascertained data, this inherent limitation should be acknowledged.^27^ Third, inclusion of self-reported health as dimension of poverty could introduce confounding in our analysis. To address this concern, we conducted a sensitivity analysis by excluding self-reported health from the index. The results (resented in Supplemental Table 2) confirmed the robustness of the positive association between multidimensional poverty and ASCVD. Additionally, sensitivity to the choice of dimensions to define multidimensional poverty and dimensional weights should be addressed, as they might influence results. The weight affixed to each dimension reflects the normative value that a deprivation in that dimension has for poverty, relative to deprivations in the other dimensions. However, universal weights don't exist, and differential weights challenge the reliability of poverty measurement. Hence, consistent with Alkire and Foster,^8^ the authors assigned all dimensions an equal weight of one-quarter for consistent population classification. Poverty literature proposes many ways to select and apply weights.^28^ Future research should investigate additional dimensions and indicators of poverty as well as optimal weights for poverty dimension in the United States, as it exceeds the scope of this paper.

## Conclusions

Individuals with ASCVD face a greater burden of multidimensional poverty than those without ASCVD. Our findings reveal that, compared to income poverty, multidimensional poverty is a stronger predictor of ASCVD. Hence, based solely on income, poverty may be insufficient for identifying individuals at a higher risk of ASCVD. Recognizing the importance of multidimensional poverty in relation to ASCVD risk can help health care professionals and policymakers in designing targeted interventions. The results also highlight the need for further research into the relationship between multidimensional poverty and health disparities, particularly in the context of ASCVD. This could unveil new insights into the complex interplay between various socioeconomic factors and cardiovascular health outcomes.

PERSPECTIVES **COMPETENCY IN SYSTEMS-BASED PRACTICE:** The study offers a comprehensive approach to assessing poverty by incorporating multiple dimensions, including income, health, health insurance, and education. This enables a more holistic understanding of the complex relationship between poverty and ASCVD risk. The utilization of the Alkire-Foster method, a robust measurement tool for multidimensional poverty, ensures the validity and reliability of the findings. This method also allows for international comparability, enhancing the study's significance and applicability across different contexts.

**TRANSLATIONAL OUTLOOK 1:** Informed intervention design: The study's findings inform the design of targeted interventions, optimizing resource allocation by focusing on high-impact poverty domains that significantly influence ASCVD risk among vulnerable populations.

**TRANSLATIONAL OUTLOOK 2:** The study's methodology and findings can serve as a baseline for future research and evaluation efforts. By tracking changes in poverty and ASCVD risk over time, researchers and policymakers can assess the effectiveness of interventions and policies aimed at reducing health disparities and improving cardiovascular health.

## Funding support and author disclosures

Dr Nasir is on the advisory board of Amgen and Novartis, and his research is partly supported by the Jerold B. Katz Academy of Translational Research. All other authors have reported that they have no relationships relevant to the contents of this paper to disclose.
