# Supplementary material for: Multidimensional Poverty and Risk of Atherosclerotic Cardiovascular Disease: A U.S. National Study
Source: JACC Adv. 2024 May 15;3(7):100928. doi: 10.1016/j.jacadv.2024.100928 (PMC11312363; doi:10.1016/j.jacadv.2024.100928)
Supplement: Supplemental Data [file mmc1.docx]

# **SUPPLEMENTAL APPENDIX**

## **Mathematical Formulation of Multidimensional Poverty Index**

The AF method first computes the proportion of people who are identified as multi-dimensionally poor, thereby producing a simple headcount ratio (incidence) of multidimensional poverty, **(*H)***. Next, average share of weighted dimensions in which poor people experience deprivation is calculated by summing deprivation scores of the poor and dividing them by the total poor population. This produces the intensity of multidimensional poverty ***(A)***. The AF Adjusted Headcount Ratio ***M_0_*** is simply a product of both poverty partial indices: ***M_0_ = H x A.***

All mathematical notation in this section follows Alkire and Foster, 2011. Let $d$ be a fixed set of dimensions to assess poverty, such that $d\mathbb{\in N}$ and $d$ can be represented by non-negative real value indicators. The achievement of person *i* in dimension *j* is denoted by $x_{ij}\in\mathbb{R}_{+}$ where *i = 1,…,n, j = 1,…,d* and ℝ_+_ is the set of non-negative real numbers. Then achievements of all people in a society are denoted by an $n x d$ dimensional achievement matrix *X*:

**Dimensions**

$$Achievement Matrix X =\left[ \begin{matrix} x_{11} & \cdots& x_{1d} \\ \vdots& \ddots& \vdots\\ x_{n1} & \cdots& x_{nd} \end{matrix} \right]$$

**People**

The d-dimensional row vector $x_{i*}$ in matrix *X* represents the achievement of any person *i* in all d dimensions for all *i = 1,…,n,*. The n-dimensional column vector $x_{*j}$ in matrix *X* represents the achievements of all n persons for a given dimension *j* for all *j = 1,…,d* in a society.

First Cutoff: Identifying Deprivations and Obtaining Deprivation Scores:

For each dimension *j*, there is a minimum threshold of achievement $z_{j}$such that if $x_{ij}< z_{j}$ then the person is deprived in dimension j. Conversely, if the person’s achievement is at least as great as the minimum threshold of achievement, i.e. $x_{ij}\geq z_{j},$ then the person is non-deprived in the dimension. Let vector $z=(z_{1},. . ., z_{d})$ contain deprivation cutoffs for all $d$ dimensions. The deprivations across dimensions may differ in relative importance. Hence, let vector $w=(w_{1},. . ., w_{d})$ contain weights or deprivation values used to indicate the relative importance of a deprivation in each dimension, where $w_{j}>0$. If normalized weights are used, then $\sum_{j} w_{j}= 1$. Alternatively, if non-normalized weights are used, $\sum_{j} w_{j}= d$

From the achievement matrix *X* and vector of dimensional deprivation cutoffs z, one can obtain a deprivation matrix $g^{0}$ such that $g_{ij}^{0}=1$ whenever $x_{ij}< z_{j}$ and $g_{ij}^{0}=0$ whenever $x_{ij}\geq z_{j}$. In other words, the deprivation matrix $g^{0}$ gives each person *i* a deprivation status value of 1 if their dimensional achievement is below the deprivation cutoff and 0 otherwise. The row vector $g_{i*}^{0}$ summarizes the deprivation status of person *i* in all d dimensions for all *i = 1,…,n*. The column vector $g_{*j}^{0}$ summarizes the deprivation status of all n persons for dimension *j* for all *j = 1,…,d* . Note that matrix $g^{0}$ is a censored matrix because achievements above the deprivation cutoffs are converted to 0.

$${Deprivation Matrix g}^{0}=\left[ \begin{matrix} g_{11}^{0} & \cdots& g_{1d}^{0} \\ \vdots& \ddots& \vdots\\ g_{n1}^{0} & \cdots& g_{nd}^{0} \end{matrix} \right]$$

Next, one can obtain an $n x d$ weighted *deprivation matrix* $ḡ^{0}=[ḡ_{ij}^{0}]$ such that $ḡ_{ij}^{0}= w_{j}$ when $x_{ij}< z_{j}$ and $ḡ_{ij}^{0}=0$ when $x_{ij}\geq z_{j}.$

$$Weighted Deprivation Matrix ḡ^{0}= w*g^{0}= \left[ \begin{matrix} ḡ_{11}^{0} & \cdots& ḡ_{1d}^{0} \\ \vdots& \ddots& \vdots\\ ḡ_{n1}^{0} & \cdots& ḡ_{nd}^{0} \end{matrix} \right]$$

The *i*^th^ row vector of matrix $ḡ^{0}$, $ḡ_{i*}^{0}$, is person *i*'’s deprivation vector. From the *deprivation matrix* $ḡ^{0}$, one can construct a column vector $c=(c_{1},. . ., c_{n})$ of weighted *deprivation scores*, such that $c_{i}= \sum_{j=1}^{d} w_{j}*{g_{ij}}^{0}= \sum_{j=1}^{d} ḡ_{ij}^{0}$ represents the sum of the weights for the dimensions in which person *i* is deprived. If $\sum_{j} w_{j}= 1$, then $0 \leq c_{i} \leq1$. A person who is non-deprived in all dimensions will have a *deprivation score* of 0. Conversely, a person who is deprived in all dimensions will have a deprivation score of 1. If $\sum_{j} w_{j}= d$, then $0 \leq c_{i} \leq d$.

Second Cutoff: Identifying the Poor:

To identify the multidimensionally poor, the AF methodology sets a *poverty cutoff* k, a minimum deprivation score a person needs in order to be identified as poor, where $0<k\leq d$. The poverty cutoff is implemented using an identification function $\rho_{k}$which is a function of each person’s achievement vector $x_{i*}$, the deprivation cutoff vector $z$, the weight vector $w$ and the poverty cutoff k. If $c_{i}\geq k$, then $\rho_{k}\left( x_{i*};z \right)=1$, otherwise, $\rho_{k}\left( x_{i*};z \right)=0$. In other words, if person *i* experiences poverty, the identification function takes a value of 1; if person *i* is non-poor, the function takes a value of 0. If the criterion for identification of multidimensionally poor is deprivation in at least on dimension ${(c}_{i}\geq0)$, then identification strategy is called union approach (Atkinson, 2003). At the other extreme, if the criterion for identification is deprivation in all dimensions ${(c}_{i}=1)$, then identification strategy is called intersection approach. Both the union and intersection approaches are special cases of identification function $\rho_{k}\left( x_{i*};z \right)$ where $k=1$ and $k=d$.

Censoring:

Using identification function $\rho_{k}\left( x_{i*};z \right)$, one can obtain censored deprivation matrix $g^{0}(k)$, where ${g_{ij}}^{0}\left( k \right)= {g_{ij}}^{0}*$ $\rho_{k}\left( x_{i*};z \right)$ for all *i* and for all *j*. If person *i* is poor i.e. $\rho_{k}\left( x_{i*};z \right)= 1$, each element of $g^{0}(k)$, ${g_{ij}}^{0}\left( k \right)$, takes value of 1. If person *i* is non-poor, i.e. $\rho_{k}\left( x_{i*};z \right)=0$, then their deprivations are censored and each element of $g^{0}(k)$, ${g_{ij}}^{0}\left( k \right)$, takes value of 0.

Similarly, one can also obtain censored deprivation score vector $c(k)$, where $c_{i}\left( k \right)= \rho_{k}\left( x_{i*};z \right)*c_{i}$ for *i = 1,…,n*. Thus, when $c_{i}\left( k \right)>k$, then $c_{i}\left( k \right)= c_{i}$, but if $c_{i}\left( k \right) <k$, $c_{i}\left( k \right)$ = 0.

Aggregation:

The Adjusted Headcount Ratio $M\text{0}$_,_ is the mean of the censored deprivation score vector:

$$M\text{0} = \mu\left( c\left( k \right) \right)=\frac{1}{n}\times\sum_{i=1}^{n} c_{i}\left( k \right)$$

$M\text{0}$ can also be written as product of two partial indices *H* and *A*, which are the incidence of poverty and the intensity of poverty. *H* is simply the **multidimensional headcount ratio**. Mathematically, $H=q/n$, where q is the number of persons identified as multidimensionally poor using the dual-cutoff counting approach. $A$ is the **average deprivation score** across poor population. $A= \sum_{i=1}^{q} c_{i}(k)/q$, where $c_{i}(k)$ is the share of possible deprivations experiences by poor person *i.* Thus, $M_{0}$ can also be expresses as:

$$M_{0}\left( X;z \right)= \mu\left( c\left( k \right) \right)=H \times A= \frac{q}{n}\times\frac{1}{q}\sum_{i=1}^{q} c_{i}\left( k \right)= \frac{1}{n}\sum_{i=1}^{n} \sum_{j=1}^{d} w_{j}g_{ij}^{0}$$

$M\text{0}$ can be interpreted two ways: as the incidence of poverty ‘adjusted’ by poverty intensity or as the aggregate deprivations experienced by the poor as a share of maximum possible range of deprivations that would occur it all members of the society were deprived in all dimensions (Alkire & Foster, 2011).

Subgroup Decompositions of M_0_:

The additive nature of $M\text{0}$ allows it to be intuitively broken across population subgroups and dimensions to monitor subgroup poverty. Suppose there are m different subgroups in a population and population share and achievement matrix of subgroup l are denoted by $v^{l}= n^{l}/n$ and $X^{l}$. Then, $M\text{0}$ can be expressed as population-share weighted sum of subgroup poverty levels.

$$M_{0}\left( X \right)= \sum_{l=1}^{m} v^{l}M_{0}(X^{l})$$

Furthermore, we can also compute the contribution of each subgroup to overall poverty as following:

$$\mathbb{D}_{l}^{0}=v^{l}\frac{M_{0}(X^{l})}{M_{0}(X)}$$

## **Supplemental Table 1:** Comparison of characteristics of participants from the National Health Interview Survey 2007-2018 included and excluded in the present analysis

|  | **Study Population** | **Excluded** |
| --- | --- | --- |
|  | **N (%)** | **N (%)** |
| **Weighted Sample** | 328,164 (91.74) | 29,550 (8.26) |
| **Age** |  |  |
| Mean (SD) | 46.3 (17.6) | 51.24 (22.0) |
| **Sex** |  |  |
| Male | 147,734 (45.0) | 12,032 (45.0) |
| Female | 180,430 (55.0) | 17,518 (55.0) |
| **Race and ethnicity** |  |  |
| Hispanic | 52,727 (16.1) | 5,007 (15.8) |
| Non-Hispanic Asian | 18,802 (5.73) | 1,805 (5.91) |
| Non-Hispanic Black | 46,103 (14.1) | 4,840 (14.2) |
| Non-Hispanic White | 206,903 (63.1) | 17,572 (63.0) |
| **Cardiovascular Risk Profile** |  |  |
| Optimal | 164,445 (55.0) | 12,934 (40.0) |
| Average | 122,731 (37.2) | 12,125 (42.1) |
| Poor | 28,503 (7.71) | 2,343 (7.93) |
| **Comorbidities** |  |  |
| 0 | 117,113 (59.1) | 8,305 (60.0) |
| 1 | 58,549 (26.8) | 4,141 (26.0) |
| ≥2 | 34,111 (14.7) | 2,419 (14.1) |
| Abbreviations: ASCVD, atherosclerotic cardiovascular disease; SD, Standard deviation.  *χ2 test (t-test for *age*): p<0.05 for covariate distribution across ASCVD and non-ASCVD subgroups, for all study variables | | |

## **Supplemental Table 2**: Sensitivity Analysis - Association between the multidimensional poverty weighted deprivation score (*c_i_*) calculated after excluding self-reported health from the index and prevalent ASCVD

|  | **Model 1^a^** | | **Model 2^b^** | | **Model 3^c^** | |
| --- | --- | --- | --- | --- | --- | --- |
| **Multidimensional Poverty** | **PR (95% CI)** | ***P* value** | **PR (95% CI)** | ***P* value** | **PR (95% CI)** | ***P* value** |
|  |  |  |  |  |  |  |
| c_i_ = 0 | Reference |  | Reference |  | Reference |  |
| c_i_ = 0.25 | 1.25 (1.21,1.29) | < .001 | 1.51 (1.46,1.55) | < .001 | 1.30 (1.24,1.35) | < .001 |
| c_i_ = 0.50 | 1.21 (1.15,1.27) | < .001 | 1.76 (1.67,1.85) | < .001 | 1.44 (1.35,1.54) | < .001 |
| c_i_ = 0.75 | 0.57 (0.49,0.66) | < .001 | 1.38 (1.20,1.60) | < .001 | 1.27 (1.04,1.54) | 0.017 |
| ^a^Model 1 = Unadjusted. ^b^Model 2 = Adjusted for age, sex and ethnicity/race. ^c^Model 3 = Adjusted for Model 2 + Cardiovascular Risk Factors Profile + Comorbidities.  Abbreviations: ASCVD, atherosclerotic cardiovascular disease; CI, confidence interval; PR, prevalence ratios; c_i_, multidimensional poverty weighted deprivation score calculated without self-reported health.  The c_i_ represents the weighted proportion of simultaneous deprivations across multiple dimensions of poverty experienced by each individual. Income, education and health insurance were used as dimensions of poverty. The c_i_ has values 0, 0.33, 0.66 and 1.00 respectively. | | | | | | |
